# Effects of the characteristic temperament of cats on the emotions and hemodynamic responses of humans

**DOI:** 10.1371/journal.pone.0235188

**Published:** 2020-06-25

**Authors:** Takumi Nagasawa, Mitsuaki Ohta, Hidehiko Uchiyama

**Affiliations:** 1 Graduate School of Agriculture Studies Tokyo University of Agriculture, Atsugi, Kanagawa, Japan; 2 Faculty of Agriculture, Tokyo University of Agriculture, Atsugi, Kanagawa, Japan; Politecnico di Milano, ITALY

## Abstract

Cats positive effects on their owners’ physiological and psychological health, including improved mood and activation of the human prefrontal cortex and inferior frontal gyrus in the brain. However, the association between the health benefits provided by cat ownership and the characteristic behaviors and reactions of cats is unclear. We recruited 29 participants to measure human prefrontal cortex activity, using functional near-infrared spectroscopy, during interactions with a cat. After the experiments, participants subjectively responded to a questionnaire regarding success rates for interactions with the cat, and completed the Self-assessment Manikin—a scale used to measure emotion. Interactions comprised eight types in four categories (touch, play, train, and feed). This study showed that interactions with a cat significantly activated the prefrontal cortex, regardless of interaction type. During training, the integral values of oxygenated hemoglobin in the left inferior frontal gyrus were the highest in all the interaction categories; however, success rates were lower than in the touch and feed interactions. Regarding the Self-assessment Manikin scores, all interaction categories showed a positive correlation between success rate and valence score, especially in the train and play interactions than in the touch and feed interactions. These results indicate that interactions with a cat activate the prefrontal cortex in humans, including the inferior frontal gyrus region. Moreover, cats’ autonomous behaviors and reactions positively influenced the participants. The characteristic temperament of cats may be a key factor influencing the health benefits of owning cats.

## Introduction

Humans throughout the world have pets, especially dogs (*Canis familiaris*) and cats (*Felis silvestris catus*), which have positive effects on the health of their owners. Growth from knowledge reported that 57% of people internationally have at least one pet (dogs: 33%, cats: 23%) [1], and 64% of such people regularly spend time with pets to maintain their physical health [2]. Previous studies also showed that companion animals can promote people’s physiological, psychological, and social health [3]. Recently, the number of pet cats has grown to exceed that of dogs in some countries [1], including Japan (dogs: 8,903,000, cats: 9,649,000) [4]. Cats have adapted to co-exist with humans and become the most popular companion animal.

Some studies have reported that owning a cat provides beneficial health effects for the owner. Cat ownership has been linked to a reduced risk for minor health problems, such as headaches and hay fever [5]. Petting a cat can decrease blood pressure and heart rate [6], and cat ownership is associated with a decreased risk of death due to myocardial infarction or cardiovascular disease [7]. Furthermore, cats can be sources of emotional support for their owners [8], and reduce negative emotions [9]. These findings indicate that cats can provide both physiological and psychological health benefits.

Recent studies have shown an association between owning pets and improved executive functions [10], such as working memory [11], which is controlled by the brain’s prefrontal cortex (PFC) [12]. Studies have shown that the PFC can be activated by petting [13] or hearing a cat [14]. In one study, Kobayashi and colleagues primarily focused on the inferior frontal gyrus (IFG) region of the PFC [13], which controls functions related to nonverbal communication, such as theory of mind [15], processing others’ facial expressions [16], and empathy [17]. Moreover, individuals with autism spectrum autism spectrum disorder (ASD) often have impaired function in the IFG region and a deficit in the mirror neuron system [18]; therefore, owning a cat could help improve nonverbal communication skills of individuals with ASD. However, previous research only focused on one source of sensory stimulation at a time (e.g., tactile [13] or auditory [14]). Moreover, these studies were not designed to focus on the everyday interactions between cats and their owners.

In households with cats, feeding, playing, and physically interacting with cats is common [19], as well as essential for building good relationships between cats and their owners. Recently, positive reinforcement training, such as clicker training, has been shown to be an effective method to improve the welfare of cats in animal shelters [20]. However, it is unclear whether there is an association between these interactions with cats and the health benefits people can experience through cat ownership.

Compared to cat ownership, the health benefits of dog ownership are more apparent. Walking a dog, one of the everyday interactions between dogs and their owners, could have various health benefits for dog owners. Walking a dog activated parasympathetic nerve activity [21], and created a habit of engaging in physical activity [22]. Moreover, walking a dog could increase one’s opportunities for social interactions with others [23]. Dog ownership has also been associated with a reduction in the risk of cardiovascular disease [24] and its associated mortality rate. Additionally, in several studies, researchers focused on dogs’ behavioral reactions during experiments. For example, Nagasawa and colleagues reported that gazing behavior from dogs increased urinary oxytocin concentrations in owner [25][26]. Another study reported that interaction with dogs could affect the concentration of hormones such as cortisol and oxytocin in their owners’ blood [27].

Through the process of domestication, dogs have learned to display obedient behaviors toward humans. The purpose of domesticating dogs was to allow them to work with humans (e.g., guarding and hunting [28]); thus, more submissive traits were chosen by artificial selection. On the contrary, cats do not typically display obedient behaviors toward humans. As cats were originally utilized for their instinctual hunting ability to decrease rodent populations [29], cats may have been domesticated by natural selection, not selective breeding [28]. Marinelli and colleagues found the factors that affect the quality of dog–owner relationships differ from those for cat–owner relationships [30], and stated the tools used to study human–animal bonds need to be customized by species. Therefore, in the study of human–cat relationships, the behaviors and reactions of a cat should be the point of focus to determine if it is the factor that leads to health benefits for cat owners.

We designed this study based on various everyday cat–human interactions, focusing on the characteristic temperament of cats during regular interactions, and examined whether the characteristic temperament of cats affects human physiological and psychological health by assessing a cat’s behavioral reactions. We hypothesized that everyday interactions with a cat activates the PFC of the human brain, including the IFG region, and affects human’s moods. Particularly, the characteristic behaviors and reactions of cats could positively influence these effects.

## Materials and methods

### Ethics statement

The experiments in this study were approved by the Human Research Ethics Committee (approval no. 1134) and Animal Experiment Ethics Committee (approval no. 1301312) at the Tokyo University of Agriculture in accordance with the World Medical Association’s Declaration of Helsinki.

### Participants and the test animal

We recruited 29 participants (10 men and 19 women) from the Tokyo University of Agriculture. Participants’ mean age was 21.17 ± 0.65 years. Sixteen participants had experience owning cats. All participants provided verbal informed consent before the experiment. No participants were allergic to cats. One spayed female cat (ragdoll breed; nine-years-old) was used in this study. The cat had always lived in the laboratory and was already trained to perform some behaviors by using positive reinforcement (e.g., raising her paws, touching humans’ hands, sitting down, turning around, lying down, etc.). Before the experiment, all participants encountered the cat while receiving an explanation of the experimental protocol; there was no person who had a specific and close relationship with the cat.

### Functional near-infrared spectroscopy (fNIRS)

**Fig 1:**
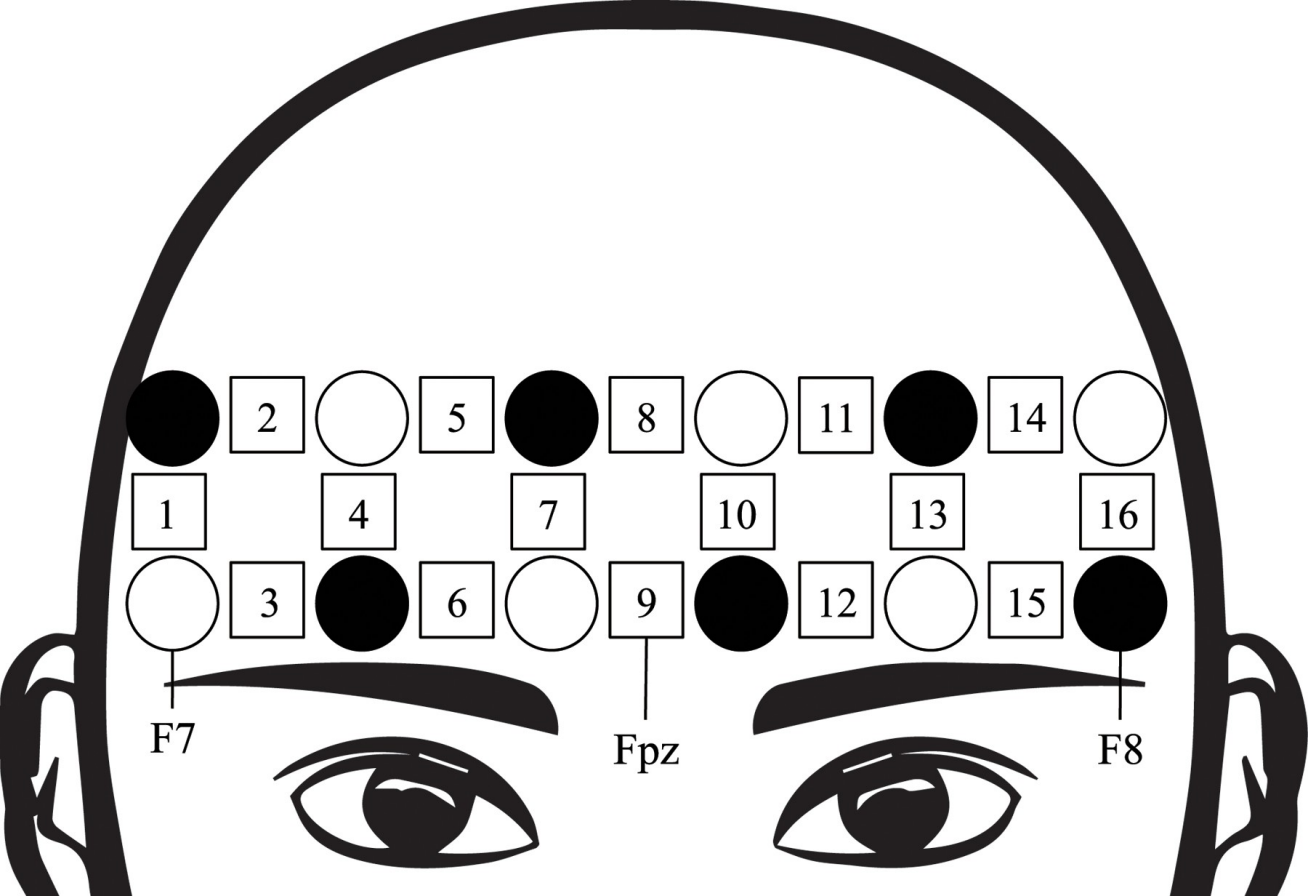
Position of OEG-SpO2 probes and channels. Black circles are illuminators. White circles are detectors. The number is the measurement region of 16 channels.

During all experimental tasks, oxygenated hemoglobin (oxy-Hb) concentrations were recorded using the fNIRS method. We used an OEG-SpO2 (Spectratech, Inc., Kanagawa, Japan). Fig 1 shows the arrangement of the apparatus. Using the basis of the International 10/20 System, the center of the probe holder was placed on Fpz, the bottom left of the corner was placed on F7, and the bottom right of the corner was placed on F8 [31]. The temporal resolution was set at 0.08 s. This fNIRS method has approximately 770 and 840 nm wavelengths as near-infrared light. This instrument consists of six illuminator probes and six detector probes. The distance between the illuminator and the detector was fixed at 3 cm.

### Self-Assessment Manikin (SAM)

**Fig 2:**
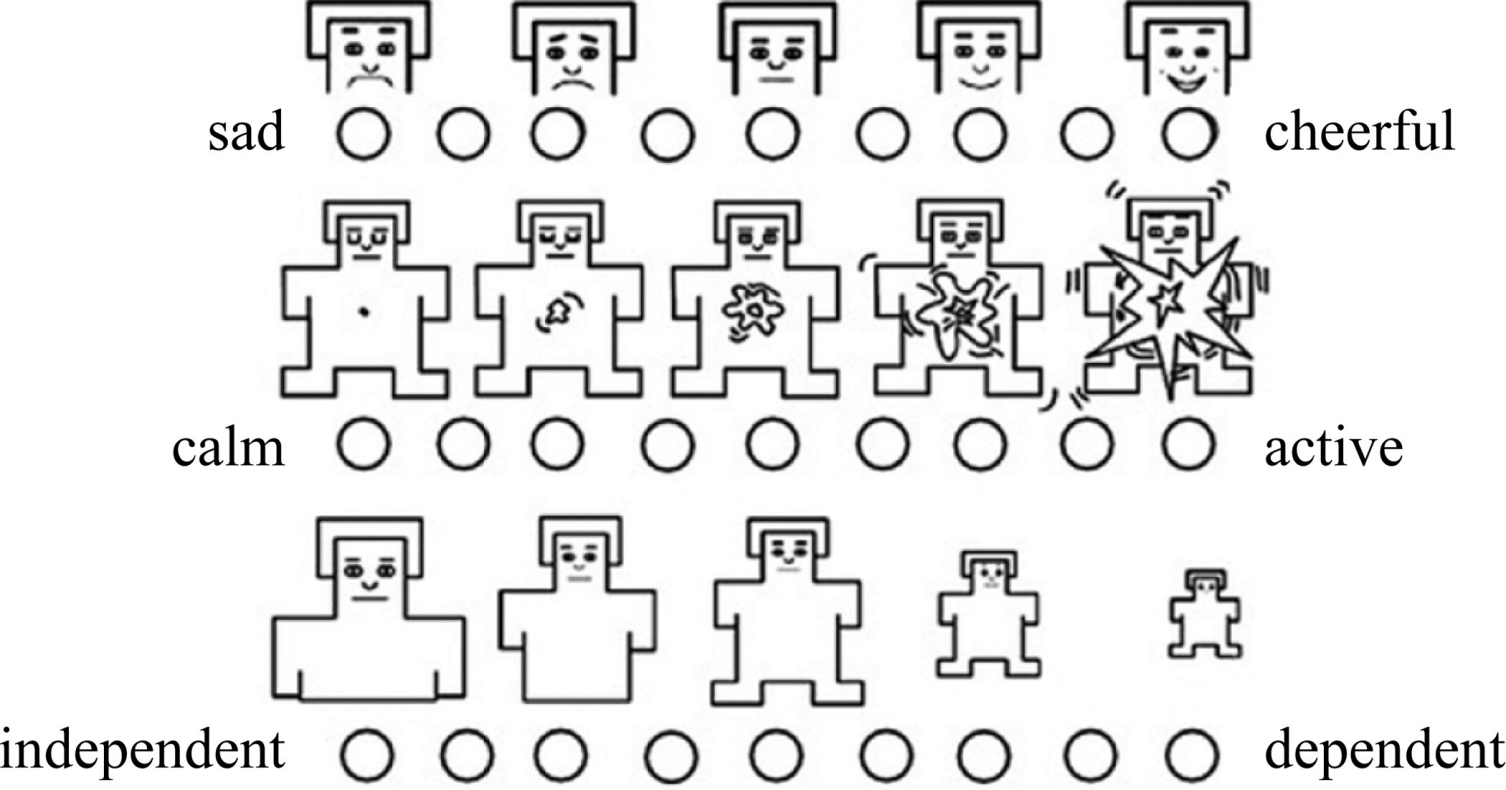
The format of the SAM.

The SAM is a nonverbal pictorial assessment method that directly and quickly measures affective responses in many contexts [32]. It comprises three dimensions (valence, arousal, and dominance) consisting of 5-panel graphic depictions, rated on a 9-point scale (see Fig 2). We focused on the valence and arousal dimensions because they are the two factors related to emotions.

### Behavioral reactions of the cat

After their interactions with the cat, participants subjectively assessed (by percentage) whether the interaction with the cat was successful (0%: *the cat did not obey* to 100%: *the cat perfectly obeyed*). Each question was arranged in an order designed to reflect the thoughts of the participants (e.g., touch interaction category: did the cat allow itself to be brushed/petted; see S1 Appendix).

### Procedure

We divided the experiment into four interaction categories (Table 1). We set two items for each interaction type. Table 1 shows the details of each interaction category: “touch” (brushing with a comb/petting by hand), “play” (with a stick/with a small rubber ball), “train” (tactile/non-tactile), and “feed” (giving food/water). The experiments consisted of one interaction item in each of the four categories.

**Table 1:** List of interaction methods used in this study.

Category	Tool	Description of the interaction
Touch	Comb	Groom the cat using a comb
	Hand	Pet the cat using hands
Play	Stick	Play with the cat using a stick
	Ball	Play with the cat using a rubber ball
Train	Type A	Command cat to turn around, lie down, and raise paw
	Type B	Command cat to give its paw and high-five
Feed	Food	Give the cat food
	Water	Give the cat water

**Fig 3:**
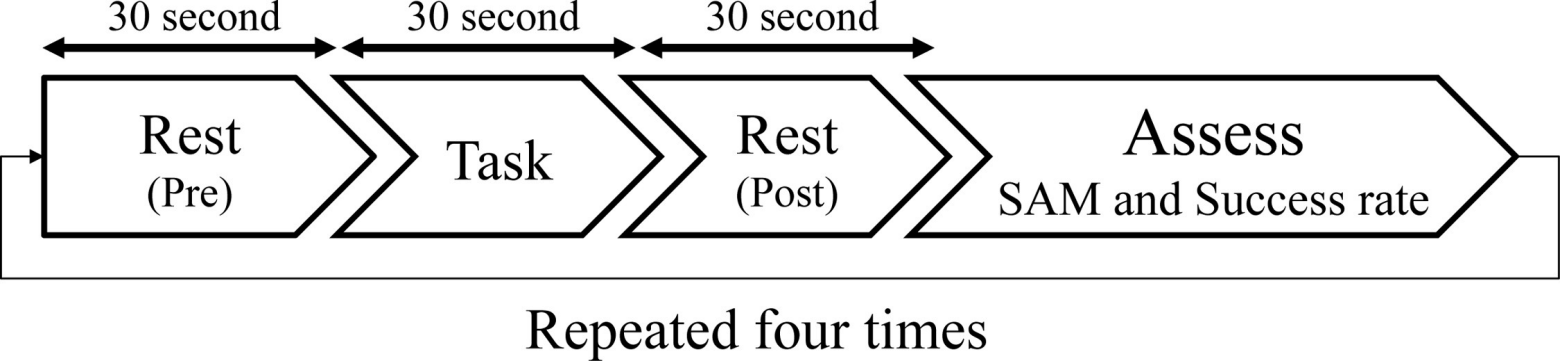
Protocol of the study experiments.

The experiment was conducted in a 3 × 3m room in the laboratory. The cat was familiar with the experimental space. The protocol of the experiments is shown in Fig 3. Participants were asked to sit in the middle of the room. For each interaction item, participants interacted with the cat for 30 seconds (task time). Before and after each interaction, participants rested for 30 seconds (pre- and post-times). During the pre- and post-times, the participants stared at a cross mark written on a paper on the wall, and repeated the Japanese vowels (/a/,/i/,/u/,/e/,/o/) in their head to stabilize their prefrontal Oxy-Hb concentrations [13]. When the task started, the experimenter put the cat in front of the participant. During all the stages of the experiment, participants were not allowed to speak to the experimenter, bow their head, or stand. However, participants were allowed to talk to the cat, but only with words related to the interaction (e.g., call the cat’s name). After the experiment, participants completed the SAM to identify their emotions and the behavior of the cat during the interactions.

### fNIRS analysis

NIRS enables us to measure signal changes in Oxy-Hb and Deoxy-Hb. The direction of changes in Oxy-Hb is always the same as that of the change in regional cerebral blood flow; however, the direction of changes in Deoxy-Hb is influenced by other factors (venous blood oxygenation and volume) [33]. Therefore, we focused only on Oxy-Hb signals. We removed the physiological fluctuations, caused by body motion and posture change, from Oxy-Hb signals using the hemodynamic modality separation system [34].To analyze participants’ Oxy-Hb signals of PFC over time during the experiments, we calculated the average value from all participants combined using the BRainAnalyzer (B.R.Systems Inc., Kanagawa, Japan). We then averaged all 16 channels in the data set.

**Fig 4:**
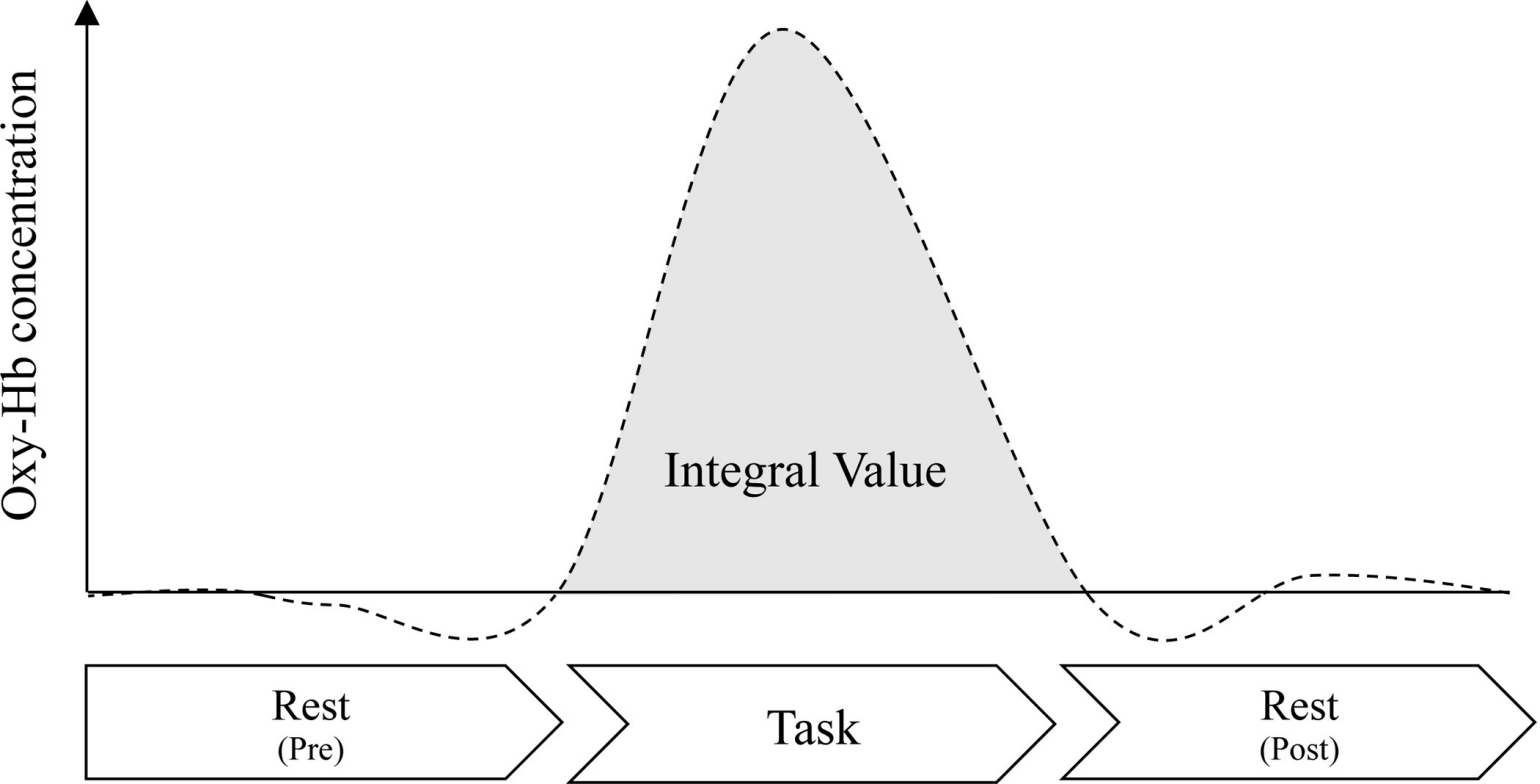
Integral value of Oxy-Hb during task time.

Based on previous study [13], to compare the degrees of activation in each interaction category, integral values of Oxy-Hb were calculated using the BRainAnalyzer (Fig 4). When we compared interaction types, we adjusted the start of the integral values to baseline. We focused on the right and left IFG regions of the brain. These regions placed F7 and F8; thus, channel 1, 2, 3, and 4 channels reflect right IFG, and 13, 14, 15, and 16 channels reflect left IFG [35].

### Statistical analysis

We determined the difference of the mean Oxy-Hb signal during rest and task time by performing an analysis of variance and a Bonferroni test as post-hoc analyses. Using the Friedman test and Scheffe’s method as post-hoc analysis, we examined the difference among four interaction categories: the integral values of the left and right IFG, participants’ mean success rate for interaction with the cat, and participants’ mean SAM score. Additionally, we compared the differences of the integral values of the left and right IFG between participants who had experience owning cats and those who did not (Welch’s *t*-tests). We assessed the correlation between success rates and the integral values of the left and right IFG and SAM score using Spearman’s rank correlation coefficient. All statistical analyses were performed using BellCurve for Excel (Social Survey Research Information Co., Ltd., Japan).

## Results

Data were analyzed from 27 participants. Two participants were excluded from analysis because one participant did not complete the experiment, and we failed to measure channel 1 of another participant.

### Sequential change of the Oxy-Hb signal

The sequential change of the Oxy-Hb signal is shown in Table 2. Significant differences in the mean Oxy-Hb concentration were observed among pre-time, task-time, and post-time (touch: F = 483.63, *p <* .01; play: F = 372.39, *p <* .01; Train: F = 509.48 *p <* .01; feed: F = 363.27, *p <* .01). A post-hoc analysis using a Bonferroni comparison indicated there were significant differences in all interaction categories: touch: pre vs. task (t = 26.82, *p <* .01), pre vs. post (t = 27.06, *p <* .01); play: pre vs. task (t = 21.76, *p <* .01), pre vs. post (t = 25.14, *p <* .01), task vs. post (t = 3.38, *p <* .01); train: pre vs. task (t = 23.82, *p <* .01), pre vs. post (t = 30.32, *p <* .01), task vs. post (t = 6.51, *p <* .01); feed: pre vs. task (t = 22.85, *p <* .01), pre vs. post (t = 23.81, *p <* .01).

**Table 2:** Mean (± SE) concentration of Oxy-Hb of PFC during Rest (pre), Task, and Rest (post).

	Rest (pre)	Task	Rest (post)
Touch	0.00 ± 0.00	0.15 ± 0.01	0.15 ± 0.01
Play	-0.01 ± 0.00	0.10 ± 0.01	0.12 ± 0.00
Train	-0.02 ± 0.00	0.15 ± 0.01	0.20 ± 0.01
Feed	0.01 ± 0.00	0.06 ± 0.00	0.06 ± 0.00

### Integral values of the IFG

**Fig 5:**
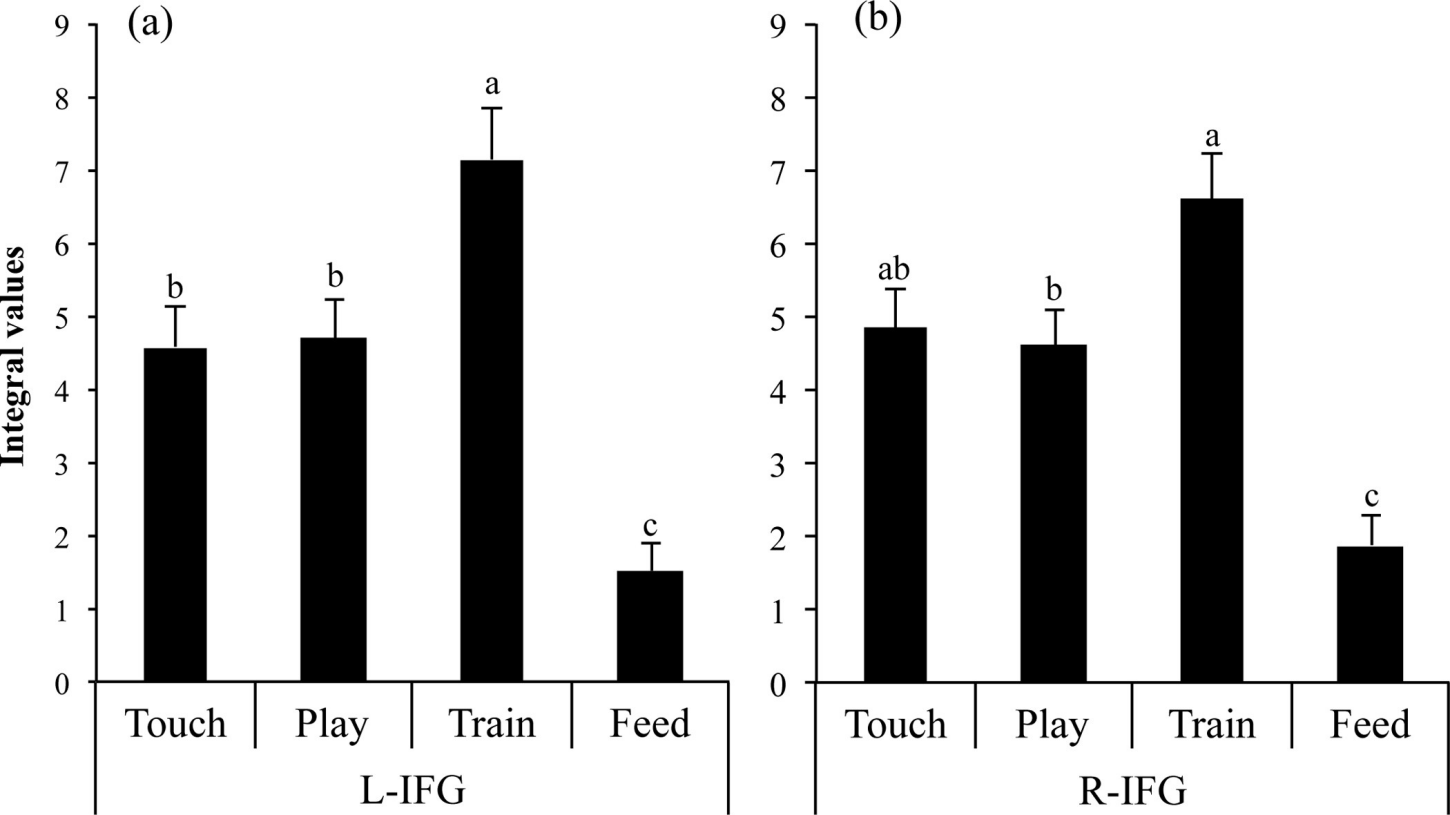
Mean integral values of the left (a) and right (b) IFG in each interaction categories. Error bars indicate SE. Different letters indicate significant differences by Scheffe’s method (*p <* .05).

Fig 5 shows the mean integral values of the right and left IFG. For both the right and left IFG, significant differences were observed among interaction categories (right IFG: X^2^ = 41.68, *p <* .01; left IFG: X^2^ = 73.11, *p <* .01).

For the right IFG, a post-hoc analysis using Scheffe’s method indicated that the feed interaction (1.88 ± 0.40) had smaller integral values than did the touch (4.84 ± 0.55, X^2^ = 16.23, *p <* .01), play (4.60 ± 0.48, X^2^ = 10.05, *p <* .05), and train (6.59 ± 0.65, X^2^ = 40.69, *p <* .01) interactions. The train interaction had higher integral values than did the play interaction (X^2^ = 10.29, *p <* .05).

For the left IFG, the feed interaction (1.52 ± 0.37) had smaller integral values than did the touch (4.58 ± 0.53, X^2^ = 27.27, *p <* .01), play (4.68 ± 0.52, X^2^ = 24.61, *p <* .01), and train (7.14 ± 0.69, X^2^ = 71.70, *p <* .01) interactions. The train interaction had higher integral values than did the touch (X^2^ = 10.53, *p <* .05) and play (X^2^ = 12.30, *p <* .01) interactions.

According to Welch’s *t*-tests, in all interaction categories, there were no significant differences in the integral values of the left and right IFG between participants who had experience owning cats and those who did not.

### SAM score

**Fig 6:**
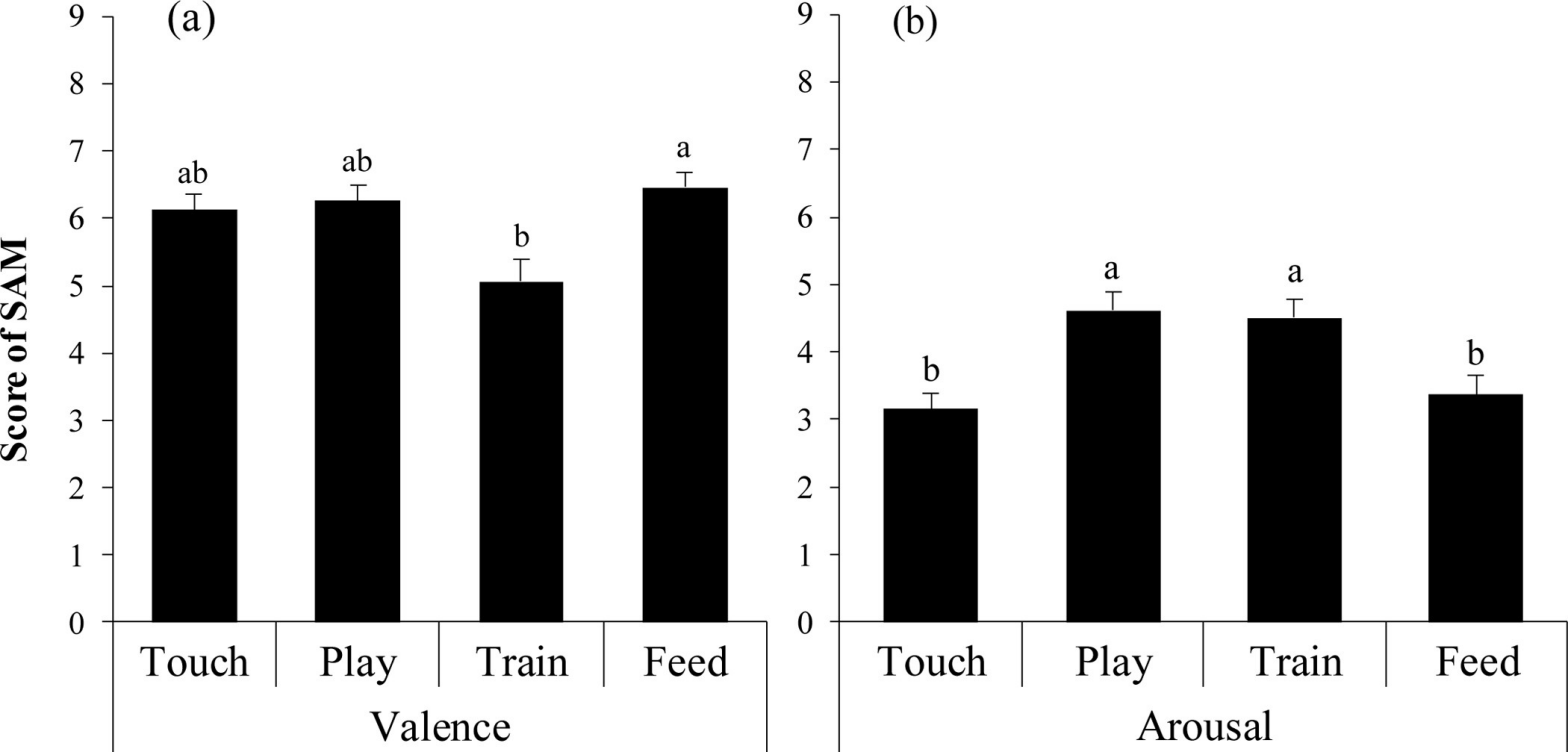
The mean SAM score among interaction categories for (a) pleasure and (b) arousal. Different letters indicate significant differences by Scheffe’s method (*p <* .05).

Valence scores showed significant differences among interaction categories (X^2^ = 9.85, *p <* .05, Fig 6A). A post-hoc analysis using Scheffe’s method indicated that the train interaction (5.06 ± 0.33) had a lower score than did the feed (6.46 ± 0.21 X^2^ = 9.34, *p <* .05) interaction.

Arousal scores also showed significant differences among interaction categories (X^2^ = 29.37, *p <* .01, Fig 6B). The play interaction (4.61 ± 0.28) had a significantly higher score than did the touch (3.15 ± 0.24, X^2^ = 22.45, *p <* .01) and feed (3.37 ± 0.29, X^2^ = 15.64, *p <* .01) interactions. The train interaction (4.50 ± 0.28) had a significantly higher score than did the touch (X^2^ = 24.12, *p <* .01) and feed (X^2^ = 17.05, *p <* .01) interactions.

### Success rate

**Fig 7:**
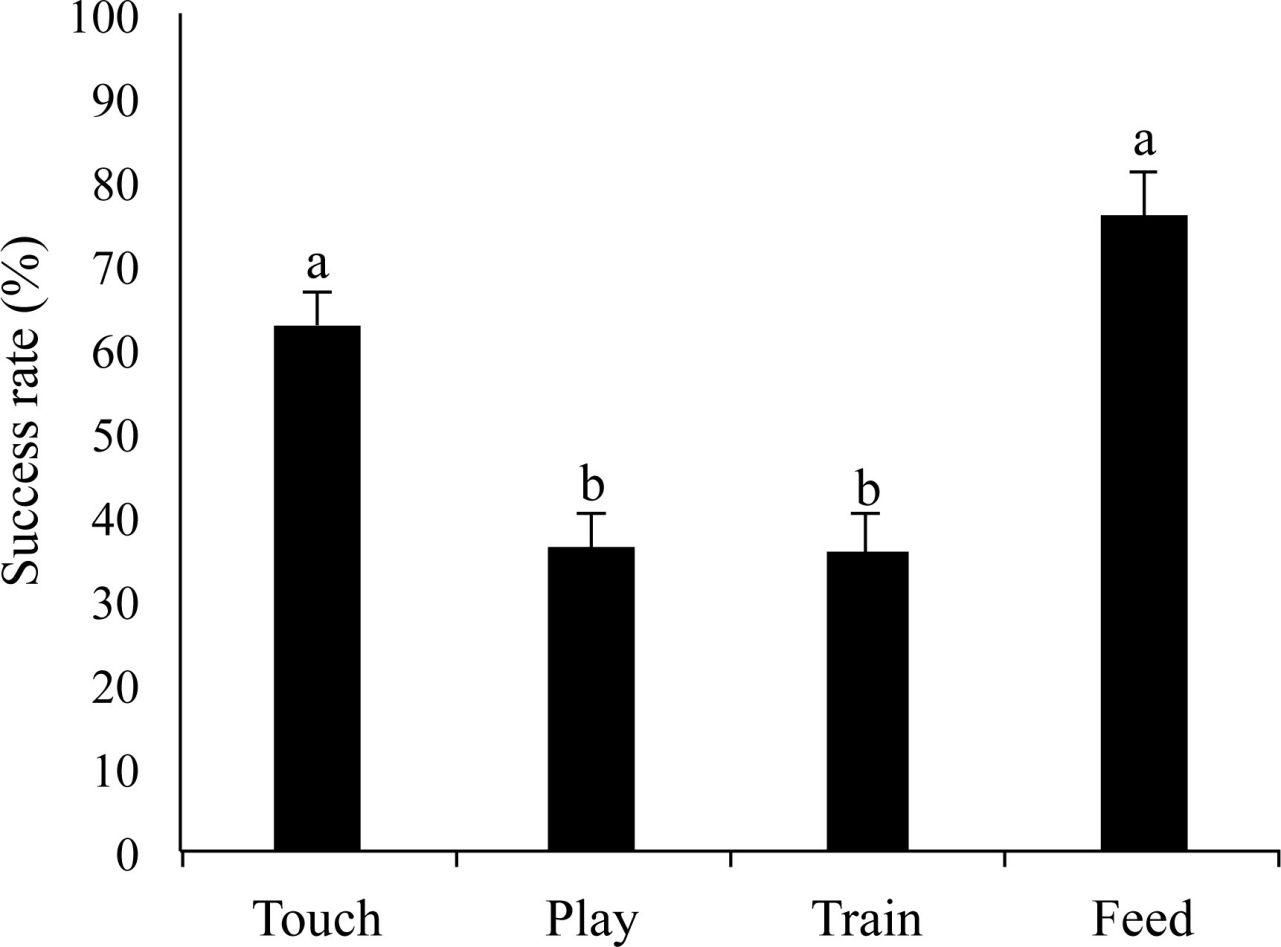
The mean success rate in all interaction categories. Error bars indicate SE. Different letters indicate significant differences by Scheffe’s method (*p <* .05).

Significant differences were observed in the success rates among interaction categories (X^2^ = 36.36, *p <* .05, Fig 7). A post-hoc analysis using Scheffe’s method indicated that the touch interaction (62.78 ± 3.93%) had a higher success rate than did the play (36.02 ± 4.55%, X^2^ = 8.56, *p <* .05) and train (35.46 ± 5.03%, X^2^ = 11.48, *p <* .01) interactions. The feed interaction (75.65 ± 5.48%) had a higher success rate than did the play (X^2^ = 24.29, *p <* .01) and train (X^2^ = 29.05, *p <* .01) interactions.

### Correlation with success rate

We analyzed the correlation between the success rate scores and the integral values of the IFG (right and left side), as well as between success rate and SAM scores individually (Table 3). The correlation between success rate and the integral values of the IFG was not significant in all interaction categories. However, SAM scores significantly correlated with success rates. For valence scores, there was positive correlation among all interaction categories (touch: r = 0.53, *p <* .05; play: r = 0.61, *p <* .01; train: r = 0.68, *p <* .01; feed: r = 0.54, *p <* .01). For arousal scores, only the play interaction positively correlated with success rate (r = 0.51, *p <* .01).

**Table 3:** Correlation coefficients between success rate and integral values of right and left IFG and SAM score.

		Touch	Play	Train	Feed
Integral values	Right	0.07	-0.10	-0.21	0.00
	Left	0.08	-0.21	-0.06	-0.15
SAM score	Valence	0.53*	0.61**	0.68**	0.55**
	Arousal	-0.03	0.51**	0.18	-0.20

*p < .05,

**p < .01.

## Discussion

### Sequential change in Oxy-Hb signal of the PFC

Interactions with a cat activated participants’ PFC, regardless of interaction type. The experiment protocol consisted of interactions typical in cat owners’ homes; therefore, this result suggested that owning a cat enhances the function of the owners’ PFC. Furthermore, PFC controls executive function [12]; therefore, interactions with a cat may improve executive function. This result is consistent with previous studies [10][11]. To our knowledge, this study may be one of the first to explain the mechanism that everyday interaction with cats enhance PFC function.

### Integral values of the IFG for the train and play interactions

We focused on the integral values of Oxy-Hb signals in left and right IFG regions. In all interactions, the integral values did not correlate with each success rate individually. However, there was a significant difference among interaction categories. The integral values of the train interaction were larger than those of the other interaction categories. Notably, in the left IFG region, the integral values of the train interaction were significantly larger than those of the other categories. The left IFG controls the mirror neuron system [36] and empathy [37]. This study suggested that performing training interactions with a cat would be an effective way to help develop these brain functions.

There are several possible reasons for significant activation of the IFG during the train interaction. First, participants might have not been accustomed to training a cat; thus, it is possible that unnatural interaction situation promoted Oxy-Hb activation for participants. Training is still not a typical interaction between a cat and its owner in general households. Nevertheless, training using clicker has recently become a standard method to improve cats’ welfare [20] and develop effective relationships between cats and humans [38]. Training a cat should be recognized as a common interaction between cats and their owners.

Second, the characteristic temperament of cats (i.e., not typically displaying obedient behavior) might have been the reason for activation of the IFG. For the train interaction, participants reported significantly lower success rates than for the feed and touch interactions. The cat frequently showed autonomous behaviors and reactions to participants owing to the independent nature of cats. Participants might try to anticipate the cat’s next action and determine how to succeed. This thinking process might have been the reason for the activation of the IFG.

The play interaction also showed a lower success rate than did the feed and touch interactions. Further, the integral values of the IFG during playing with the cat were larger than those during feeding. In the play interaction, it was difficult for participants to attract the cat to play. As with the train interaction, participants might think about the way to succeed in this interaction.

The thinking processes used during the train and play interactions related to fundamental nonverbal communication skills necessary not only for interactions between people and animals but also for interpersonal interactions. Therefore, the train and play interactions with a cat, which induce the activation of the IFG, have potential to treat individuals with ASD, which have impaired function in the IFG region [18]. Previous studies showed that interactions with an animal can improve the social communication skills of children with ASD [39][40]. Although much of the previous research has been performed using therapy dogs [41], a few studies have posited that cats can also be useful for therapy with people with ASD [42][43]. However, the mechanism was still unclear.

It is frequently difficult to speculate on cats’ behaviors, even for their owners. The behaviors and temperament of cats, such as independence, is a unique trait compared to dogs. As cat domestication was shorter than for dogs, and may not even be complete [28], the genes of domestic cats are not distinct from those of wild cats [44]; thus, even household cats frequently display autonomous behaviors like wild animals. The present results suggest that cats’ unique behaviors and reactions are the key factors explaining the mechanism underlying the health benefits that cats can provide to individuals with ASD. However, this study targeted healthy participants, not those with ASD; therefore, further studies are needed to determine whether cats positively effects the treatment of individuals with ASD.

### Integral values of the IFG for the feed and touch interactions

During the feed interaction, the integral values were significantly less compared to the other interaction types; however, the success rate was higher than in the train and play interactions. Since feeding is the most fundamental interaction between a human and an animal, the cat relatively obeyed participants during the feed interaction. Participants may have felt it was easy to speculate on the cat’s behavioral reactions during the feed interaction; therefore, the IFG region was not activated.

As with feeding, tactile communication with a cat is a central interaction between a cat and its owner. In this study, the touch interaction showed a higher success rate than either the train or the play interaction; however, the integral values of the IFG were larger than during the feed interaction. This could be the result of tactile stimulation. A previous study showed that the IFG region was activated by touching a cat [13], which is consistent with the findings of this study. Therefore, the current results might show that tactile stimuli, which occur through interaction with a cat, affect IFG activation.

### SAM

Valence scores from the SAM significantly positively correlated with success rates. The valence dimension in the SAM is the measurement of emotions, such as happiness and satisfaction [32]. In this study, participants felt positive emotion when the cat obeyed them. Moreover, the train and play interactions, which had a significantly lower success rate than the feed and touch interactions, showed a relatively higher correlation coefficient than the feed and touch interactions. Therefore, the present result indicates that the lower the success rate of interaction with a cat, the more likely positive emotions of the participants occurred when the interaction succeeds. As mentioned above, cats and dogs have different temperaments, and cats frequently showed autonomous behavior and reaction for their owners. These characteristic temperaments of cats may be the key factor to enhance human psychological status.

During the play interaction, only the arousal score for the SAM significantly positively correlated with success rates. The arousal dimension in the SAM is the measurement of emotions such as excitement [32]. In the play interaction, the success meant the cat responded to the cat toys using its paws. It is possible that the movement of the cat increased the arousal of the participants. Previous studies claimed that the arousal response is related to enhanced cognitive function [45]. Additionally, exercise, which increases arousal, also improves executive function [46][47]. Therefore, playing with a cat may promotes the development of human cognitive function. Furthermore, 90% of cat owners play with their cats at least once per day [48]; thus, play with cats is a common interaction for their owners. The results of the current study may show the mechanism of an association between owning pets and improved executive functions.

### Limitations

This study had several limitations. First, we used a laboratory cat, not a house cat. This was because of the difficulty of conducting this experiment in cat owners’ homes. Domestic cats are territorial animals [49], and would not behave typically with their owner if an unfamiliar person and apparatus were to be in their territory. Thus, we utilized a laboratory cat. However, the cat had been raised in the laboratory like as a house cat; therefore, the cat had the characteristic temperament of a house cat.

Second, during the experiments, only participants could initiate an interaction, not the cat. Specifically, in the touch interactions, we requested that participants pet the cat. However, cats often display allogrooming (i.e., groom other cats using their tongue) and allorubbing (i.e., rubbing their head and tail toward other cats) behaviors toward humans [50]. If interactions between participants and the cat had been mutual, the results may have varied. In future studies, researchers should design a protocol that allows for free and mutual interactions between cats and participants.

Third, we used Bonferroni’s and Scheffe’s methods for post-hoc analyses; although, we did not use a false discovery rate approach. Therefore, further studies should use false discovery rate to control the proportion of false positives among channels that are significantly detected.

## Conclusions

Our findings indicated that everyday interaction with a cat can activate a person’s PFC, including the IFG region, regardless of the type of interaction. Moreover, during training interactions, the cat often disobeyed the participants, which elicited significant IFG activation. Valence scores of participants positively correlated with the success rates for interactions with the cat; especially, the train and play interactions, which had significantly lower success rates than did the touch and feed interactions, and a high correlation coefficient.

This study showed that the autonomous behaviors and reactions of a cat influenced the physiological and psychological states of people; therefore, the characteristic temperament of a cat may be the key factor to the mechanism underlying the positive health effects gained through cat ownership.

## Supporting information

S1 Appendix Questionnaire for behavioral reactions of the cat. (DOCX)
