# Supplementary material for: Effects of the characteristic temperament of cats on the emotions and hemodynamic responses of humans
Source: PLoS One. 2020 Jun 25;15(6):e0235188. doi: 10.1371/journal.pone.0235188 (PMC7316254; doi:10.1371/journal.pone.0235188)
Supplement: S1 Appendix — (DOCX) [file pone.0235188.s001.docx]

**S1 Appendix**

**Japanese version of the questionnaire**

[接触] 猫はブラッシングされるのを許してくれたか？／猫は撫でられるのを許してくれたか？ 　　　　%

[遊び] 猫は遊んでくれたか？ 　　　　%

[命令] 猫は命令に従ってくれたか？ 　　　　%

[給餌] 猫は餌を食べてくれたか？ ／ 水を飲んでくれたか？ 　　　　%

**English version of the questionnaire**

[Touch] Did the cat allows itself to be brushed/touched? 　　　　%

[Play] Did the cat play? 　　　　%

[Train] Did the cat obey your commands? 　　　　%

[Feed] Did the cat eat cat food/drink water? 　　　　%
